# Supplementary material for: Structure and dynamics of Toll immunoreceptor activation in the mosquito Aedes aegypti
Source: Nat Commun. 2022 Aug 30;13:5110. doi: 10.1038/s41467-022-32690-6 (PMC9427763; doi:10.1038/s41467-022-32690-6)
Supplement: Supplementary file 3 — Reporting Summary [file 41467_2022_32690_MOESM3_ESM.pdf]

nature portfolio

Corresponding author(s): Monique Gangloff

Last updated by author(s): Jul 25, 2022

Reporting Summary

Nature Portfolio wishes to improve the reproducibility of the work that we publish. This form provides structure for consistency and transparency in reporting. For further information on Nature Portfolio policies, see our Editorial Policies and the Editorial Policy Checklist.

Statistics

For all statistical analyses, confirm that the following items are present in the figure legend, table legend, main text, or Methods section.

n/a	Confirmed	
☐	☒	The exact sample size (*n*) for each experimental group/condition, given as a discrete number and unit of measurement
☐	☒	A statement on whether measurements were taken from distinct samples or whether the same sample was measured repeatedly
☐	☒	The statistical test(s) used AND whether they are one- or two-sided<br>*Only common tests should be described solely by name; describe more complex techniques in the Methods section.*
☐	☒	A description of all covariates tested
☒	☐	A description of any assumptions or corrections, such as tests of normality and adjustment for multiple comparisons
☐	☒	A full description of the statistical parameters including central tendency (e.g. means) or other basic estimates (e.g. regression coefficient) AND variation (e.g. standard deviation) or associated estimates of uncertainty (e.g. confidence intervals)
☐	☒	For null hypothesis testing, the test statistic (e.g. *F*, *t*, *r*) with confidence intervals, effect sizes, degrees of freedom and *P* value noted<br>*Give P values as exact values whenever suitable.*
☒	☐	For Bayesian analysis, information on the choice of priors and Markov chain Monte Carlo settings
☒	☐	For hierarchical and complex designs, identification of the appropriate level for tests and full reporting of outcomes
☒	☐	Estimates of effect sizes (e.g. Cohen's *d*, Pearson's *r*), indicating how they were calculated

Our web collection on statistics for biologists contains articles on many of the points above.

Software and code

Policy information about availability of computer code

Data collection	EPU v2.10
Data analysis	WARP 1.0.9, CryoSPARC v3.3.1, Coot 0.9, Phenix 1.19, PyMol v2.1.1, GraphPad Prism v9.3.1, Jalview 2.11.1.0, Sedfit 16.1c, Sednterp 2012.08.23, ASTRA 6.2, ATSAS 2.8.3, ScAtter 3.1, AcquireMP 2.2.0, DiscoverMP version 2.2.0, Modeller 9.24, Cufflinks 2.2.1, CummeRbund 2.8.2, Panther 16.0, R software version 2.28.

For manuscripts utilizing custom algorithms or software that are central to the research but not yet described in published literature, software must be made available to editors and reviewers. We strongly encourage code deposition in a community repository (e.g. GitHub). See the Nature Portfolio guidelines for submitting code & software for further information.

Data

Policy information about availability of data

All manuscripts must include a data availability statement. This statement should provide the following information, where applicable:

- Accession codes, unique identifiers, or web links for publicly available datasets
- A description of any restrictions on data availability
- For clinical datasets or third party data, please ensure that the statement adheres to our policy

Sequences are available from Vectorbase for Toll1A under the identifier: AAEL026297 [https://vectorbase.org/vectorbase/app/record/gene/AAEL026297]; Toll5A: AAEL007619 [https://vectorbase.org/vectorbase/app/record/gene/AAEL007619]; Spz1C: AAEL013433 [https://vectorbase.org/vectorbase/app/record/gene/AAEL013433]; and SpzX: AAEL013434 [https://vectorbase.org/vectorbase/app/record/gene/AAEL013434]. Spz5: AAEL001929 [https://vectorbase.org/vectorbase/

app/record/gene/AAEL001929]. Spz1A identifier AAEL000499-PA is obsolete. However, UniProt accession code for Spz1A is Q17P53 [https://www.uniprot.org/uniprotkb/Q17P53/entry]; Spz1C: Q16J57 [https://www.uniprot.org/uniprotkb/Q16J57/entry]; SpzX; A0A6I8TFH1 [https://www.uniprot.org/uniprotkb/A0A6I8TFH1/entry]; Spz5: Q17JP7 [https://www.uniprot.org/uniprotkb/Q17JP7/entry]; Toll1A: A0A6I8U6W1 [https://www.uniprot.org/uniprotkb/A0A6I8U6W1/entry]; Toll5A: A0A6I8TEX2 [https://www.uniprot.org/uniprotkb/A0A6I8TEX2/entry].

The RNA-seq data for this study are publicly available through the European Nucleotide Archive under accession number PRJEB50861 [https://www.ebi.ac.uk/ena/browser/view/PRJEB50861]. SAXS data were deposited at the Small Angle Scattering database with accession numbers SASDKX8 for Toll5A alone [https://www.sasbdb.org/data/SASDKX8] and, SASDKY8 for Toll5A with Spz1C [https://www.sasbdb.org/data/SASDKY8], respectively. The cryo-EM 3D maps corresponding to the homodimer, the ligated heterodimer and heterotrimer were deposited in EMDB database with accession codes EMD-11984 [https://www.ebi.ac.uk/emdb/EMD-11984]; EMD-11982 [https://www.ebi.ac.uk/emdb/EMD-11982] and EMD-11983 [https://www.ebi.ac.uk/emdb/EMD-11983], respectively. The corresponding atomic models were deposited in PDB with accession codes 7B1D [http://doi.org/10.2210/pdb7B1D/pdb], 7B1B [http://doi.org/10.2210/pdb7B1B/pdb] and 7B1C [http://doi.org/10.2210/pdb7B1C/pdb], respectively.

There are no restrictions on data availability.

Human research participants

Policy information about studies involving human research participants and Sex and Gender in Research.

Reporting on sex and gender	Not applicable
Population characteristics	Not applicable
Recruitment	Not applicable
Ethics oversight	Not applicable

Note that full information on the approval of the study protocol must also be provided in the manuscript.

Field-specific reporting

Please select the one below that is the best fit for your research. If you are not sure, read the appropriate sections before making your selection.

[x] Life sciences [ ] Behavioural & social sciences [ ] Ecological, evolutionary & environmental sciences

For a reference copy of the document with all sections, see nature.com/documents/nr-reporting-summary-flat.pdf

Life sciences study design

All studies must disclose on these points even when the disclosure is negative.

Sample size	Samples sizes for qPCR were independent biological triplicates with no sample-size calculation performed beforehand. For Spaeztle1C dose-response curve in Aag2 cells, sample size was determined based on consistency and variability of the data with up to 36 repeats. For other methods, number of sample size was determined following method's guidelines.
Data exclusions	No data was excluded for the analysis.
Replication	Experiments were performed on independent triplicates unless stated otherwise.
Randomization	No randomization was necessary in any of the experiments presented as none involve any case-control cohort experiments.
Blinding	No blinding was necessary in any of our experiments as our study did not involve targeted deletion of any factor in cells, but addition instead. All data were analysis in an unbiased fashion.

Reporting for specific materials, systems and methods

We require information from authors about some types of materials, experimental systems and methods used in many studies. Here, indicate whether each material, system or method listed is relevant to your study. If you are not sure if a list item applies to your research, read the appropriate section before selecting a response.

Materials & experimental systems

n/a	Involved in the study
☒	☐ Antibodies
☐	☒ Eukaryotic cell lines
☒	☐ Palaeontology and archaeology
☒	☐ Animals and other organisms
☒	☐ Clinical data
☒	☐ Dual use research of concern

Methods

n/a	Involved in the study
☒	☐ ChIP-seq
☒	☐ Flow cytometry
☒	☐ MRI-based neuroimaging

Eukaryotic cell lines

Policy information about cell lines and Sex and Gender in Research

Cell line source(s)	Aag2 Cell line (RRID: CVCL_Z617): Center for Virus Research; Glasgow.<br>SF9 Cells: ThermoFischer Scientific (Cat. No. 12659017).<br>Schneider 2 cells (RRID:CVCL_Z232): ThermoFisher Scientific (Cat. No. R69007).
Authentication	Aag2 cells were further authenticated by sequence analysis. S2 and Sf9 cells were used for protein expression so authentication not relevant
Mycoplasma contamination	All cells tested negative for mycoplasma contamination.
Commonly misidentified lines<br>(See ICLAC register)	None
